# Grapevine plantlets respond to different monochromatic lights by tuning photosynthesis and carbon allocation

**DOI:** 10.1093/hr/uhad160

**Published:** 2023-08-08

**Authors:** Menglong Liu, Yan Zhao, Peige Fan, Junhua Kong, Yongjian Wang, Xiaobo Xu, Meilong Xu, Lijun Wang, Shaohua Li, Zhenchang Liang, Wei Duan, Zhanwu Dai

**Affiliations:** State Key Laboratory of Plant Diversity and Specialty Crops, Beijing Key Laboratory of Grape Sciences and Enology, Institute of Botany, The Chinese Academy of Sciences, Beijing, 100093, China; China National Botanical Garden, Beijing 100093, China; University of Chinese Academy of Sciences, Beijing 100049, China; State Key Laboratory of Plant Diversity and Specialty Crops, Beijing Key Laboratory of Grape Sciences and Enology, Institute of Botany, The Chinese Academy of Sciences, Beijing, 100093, China; China National Botanical Garden, Beijing 100093, China; University of Chinese Academy of Sciences, Beijing 100049, China; State Key Laboratory of Plant Diversity and Specialty Crops, Beijing Key Laboratory of Grape Sciences and Enology, Institute of Botany, The Chinese Academy of Sciences, Beijing, 100093, China; China National Botanical Garden, Beijing 100093, China; University of Chinese Academy of Sciences, Beijing 100049, China; State Key Laboratory of Plant Diversity and Specialty Crops, Beijing Key Laboratory of Grape Sciences and Enology, Institute of Botany, The Chinese Academy of Sciences, Beijing, 100093, China; China National Botanical Garden, Beijing 100093, China; State Key Laboratory of Plant Diversity and Specialty Crops, Beijing Key Laboratory of Grape Sciences and Enology, Institute of Botany, The Chinese Academy of Sciences, Beijing, 100093, China; China National Botanical Garden, Beijing 100093, China; State Key Laboratory of Plant Diversity and Specialty Crops, Beijing Key Laboratory of Grape Sciences and Enology, Institute of Botany, The Chinese Academy of Sciences, Beijing, 100093, China; China National Botanical Garden, Beijing 100093, China; University of Chinese Academy of Sciences, Beijing 100049, China; Ningxia Horticulture Research Institute, Ningxia Academy of Agricultural and Forestry Sciences, Yinchuan 750002, China; State Key Laboratory of Plant Diversity and Specialty Crops, Beijing Key Laboratory of Grape Sciences and Enology, Institute of Botany, The Chinese Academy of Sciences, Beijing, 100093, China; China National Botanical Garden, Beijing 100093, China; University of Chinese Academy of Sciences, Beijing 100049, China; State Key Laboratory of Plant Diversity and Specialty Crops, Beijing Key Laboratory of Grape Sciences and Enology, Institute of Botany, The Chinese Academy of Sciences, Beijing, 100093, China; China National Botanical Garden, Beijing 100093, China; University of Chinese Academy of Sciences, Beijing 100049, China; State Key Laboratory of Plant Diversity and Specialty Crops, Beijing Key Laboratory of Grape Sciences and Enology, Institute of Botany, The Chinese Academy of Sciences, Beijing, 100093, China; China National Botanical Garden, Beijing 100093, China; University of Chinese Academy of Sciences, Beijing 100049, China; State Key Laboratory of Plant Diversity and Specialty Crops, Beijing Key Laboratory of Grape Sciences and Enology, Institute of Botany, The Chinese Academy of Sciences, Beijing, 100093, China; China National Botanical Garden, Beijing 100093, China; State Key Laboratory of Plant Diversity and Specialty Crops, Beijing Key Laboratory of Grape Sciences and Enology, Institute of Botany, The Chinese Academy of Sciences, Beijing, 100093, China; China National Botanical Garden, Beijing 100093, China; University of Chinese Academy of Sciences, Beijing 100049, China

## Abstract

The quality of planting materials is the foundation for productivity, longevity, and berry quality of perennial grapevines with a long lifespan. Manipulating the nursery light spectrum may speed up the production of healthy and high-quality planting vines but the underlying mechanisms remain elusive. Herein, the effects of different monochromatic lights (green, blue, and red) on grapevine growth, leaf photosynthesis, whole-plant carbon allocation, and transcriptome reprograming were investigated with white light as control. Results showed that blue and red lights were favorable for plantlet growth in comparison with white light. Blue light repressed excessive growth, significantly increased the maximum net photosynthetic rate (Pn) of leaves by 39.58% and leaf specific weight by 38.29%. Red light increased the dry weight of the stem by 53.60%, the starch content of the leaf by 53.63%, and the sucrose content of the stem by 230%. Green light reduced all photosynthetic indexes of the grape plantlet. Photosynthetic photon flux density (PPFD)/Ci–Pn curves indicated that blue light affected photosynthetic rate depending on the light intensity and CO_2_ concentration. RNA-seq analysis of different organs (leaf, stem, and root) revealed a systematic transcriptome remodeling and *VvCOP1* (CONSTITUTIVELY PHOTOMORPHOGENIC 1), *VvHY5* (ELONGATED HYPOCOTYL5), *VvHYH* (HY5 HOMOLOG*)*, *VvELIP* (early light-induced protein) and *VvPIF3* (PHYTOCHROME INTERACTING FACTOR 3) may play important roles in this shoot-to-root signaling. Furthermore, the correlation network between differential expression genes and physiological traits indicated that *VvpsbS* (photosystem II subunit S), *Vvpsb28* (photosystem II subunit 28), *VvHYH*, *VvSUS4* (sucrose synthase 4), and *VvALDA* (fructose-bisphosphate aldolase) were pertinent candidate genes in responses to different light qualities. Our results provide a foundation for optimizing the light recipe of grape plantlets and strengthen the understanding of light signaling and carbon metabolism under different monochromatic lights.

## Introduction

Grape is an important fruit consumed worldwide and products from grape processing, such as raisins and wine, also have high economic value. Wine grapes have a very long production lifespan of 30–50 years, but the losses caused by the failure of poor establishment and decline of newly planted vines have become a significant but often unrecognized burden for the wine industry [1]. Hence, it is vital to establish a standardized and highly efficient vine nursery that can supply planting materials that are healthy and uniform [2]. The growth of vine planting material is affected by many factors, among which light plays an important role. In recent years, ‘speed breeding’ has been proposed to accelerate plant growth and development by extending the photoperiod (22 h light/2 h dark) and optimizing the light recipe in fully enclosed, controlled-environment growth chambers, demonstrating that speed breeding in fully controlled conditions can accelerate plant development [2, 3], which provides clues for increasing the rate of propagation of virus-free and high-quality planting grapevines.

The development of LED lighting technology, allowing fine tuning of light spectra, has enabled research on and application of light quality in enhancing plant growth [4]. Moreover, instead of using a one-size-fits-all approach, lighting recipes have been determined for different plant species [3]. The phytochromes are selective for absorption spectra, with chlorophyll A showing maximum absorption at 430 and 662 nm, chlorophyll B at 453 and 642 nm, and carotene at 430 and 490 nm [5]. Therefore, the regulation of plant morphogenesis, photosynthesis, and energy metabolism varies among different monochromatic lights with distinct wavelengths of photons [6]. Moreover, a given monochromatic light often shows species-dependent or even contrasting effects on plant growth and crop quality. For instance, blue light has been reported to promote the growth and development of potato (*Solanum tuberosum* L.) [7] and cabbage (*Brassica rapa* subsp*. pekinensis*) [8], while other studies also showed blue light decreased leaf mass in Ambrozja dill (*Anethum graveolens* L.) [9], rice [10], and lamb’s lettuce and garden rocket [11] when compared with white or red light. Moreover, paradoxical results also occurred in plant photosynthesis performance. Blue light enhanced leaf stomatal conductance (*g*_s_) and total and initial rubisco activities in young cucumber [12], but decreased net CO_2_ assimilation rate of fully expanded leaves in sweet pepper (*Capsicum annuum* L.) [13]. Besides, the potential effects of red light on plant growth and photomorphogenesis have also been intensively studied [14–17]. It was reported that red light can increase the net photosynthesis rate (Np) in grapes [17] and rice [18]. Furthermore, application of red light also promoted leaf growth and stem development in cucumber [14] and grapes [15], while other studies showed red light negatively regulated leaf growth of *Alternanthera brasiliana* Kuntze, including specific leaf mass, leaf thickness, and leaf density [16]. In addition, it is reported that monochromatic green light decreased the net photosynthetic rate of onions (*Allium fistulosum* L.) [19], but increased the leaf area of sweet pepper (*C. annuum* L.) [20] and root growth of lettuce (*Lactuca sativa* L.) [21], when compared with that in white light. In short, these species-dependent and controversial effects of monochromatic light on plant growth and quality guarantee the requirement of further comprehensive investigations on the underlying mechanisms.

To determine the appropriate light recipe, it is essential to analyze the response of plant growth and development to different light qualities at the molecular level. Photosynthesis is the most efficient light energy converter in nature, with quantum efficiency close to 1 [22]. The photosynthetic system includes a cytochrome *b6f* complex (cyt *b6f*), an ATP synthase, and two photosystems (photosystem I and photosystem II, the so-called PSI and PSII, respectively) for absorbing the light. The two photosystems both have a reaction center where the photochemical reactions occur, and an antenna system responsible for light harvesting. The reaction center is the key element of the photosystem; it is composed of many subunits, of which D1 (encoding by the *psbA* gene) and D2 (encoded by the *psbD* gene) form the core skeleton of the reaction center in PSII, while PsaA and PsaB subunits form a heterodimer that binds many cofactors in PSI [23]. Antenna proteins mainly serve to harvest solar energy efficiently and then transfer it to the photosystem reaction centers. PSII includes a trimeric LHCII and three monomeric proteins (Lhcb4, Lhcb5, and Lhcb6), while the antenna of PSI includes two heterodimers composed of Lhca1/Lhca4 and Lhca2/Lhca3 [24]. Although the structure and function of proteins in the photosystem have been extensively explored, the responses of their encoding genes to different monochromatic lights remain largely unexplored.

Light not only provides energy for photosynthesis but is also an important signal for regulating plant growth and morphology [25]. In *Arabidopsis*, most photoreceptors for lights of different wavelengths have been identified. UVR8 (UV RESISTANCE LOCUS8) was reported to be the UV-B photoreceptor, CRYs (cryptochromes) and PHOTs (phototropins) are blue light photoreceptors, and PHYs (photopigments) are receptors for red and far-red light [26]. In the light signaling network, COP1, HY5/HYH, and PIFs have been characterized as the master transcription factors. COP1 (CONSTITUTIVELY PHOTOMORPHOGENIC 1) is an E3 ubiquitin ligase that regulated photomorphogenesis, shade avoidance, and flower development together with photoreceptors [25]. In the dark, COP1 interacts with SPAs and E3 ubiquitin ligase complexes to form polyubiquitinated positive regulators of light signaling that contribute to 26S proteasomal degradation [27]. The substrates of this process are mainly transcription factors, including HY5 (ELONGATED HYPOCOTYL5) and its homologs HYH, HFR1 (LONG HYPOCOYL), and CO (COSTANS). HY5 belongs to the basic leucine zipper (bZIP) family, which has been proven to respond to brassinosteroids (BRs) and gibberellin (GA) and then identified as a key point that coordinates the hormone and light signaling [26]. PIFs belong to the bHLH family, and they function as a molecular regulatory hub that monitors light, temperature, and hormone signaling [28]. However, how these light signaling players respond to different monochromatic lights is not fully explored.

The emergence of next-generation technology has facilitated the systematic monitoring of cellular responses in the transcriptome, but few studies have applied transcriptome analysis in light responses. In the grape plantlet, red and green light altered the expression levels of genes belonging to the shade-avoidance syndrome, such as auxin-repressed protein, xyloglucan endotransglucosylase/hydrolase, and histones H1, H2A, H2B, H3, and H4 in leaves [29]. In rapeseed (*Brassica napus* L.), combined light treatments (red and blue LEDs with supplementary yellow, green, or white light) induced the expression of genes associated with light stimulus and high light response, leaf development, and carbohydrate synthesis. Moreover, a recent study found the distinction of the growth and development of root and shoot under RB (mixed red and blue) light could be mediated by GA and IAA (indole-3-acetic acid) synthesis [30].

Nevertheless, current omics studies have mainly focused on the above-ground organs, such as fruits and leaves, but have neglected roots as an equally important compartment. Combining the responses of both above- and underground organs to different light spectra will provide valuable information about whether the light signal exerts functions locally or systemically with long-distance signaling transduction [31].

To date, the effects of different light qualities on plant performance have been studied extensively, but systematic and comprehensive research in grape is still limited, particularly for the underlying molecular mechanisms of different light qualities regulating photosynthesis and carbon metabolism. How light quality affects carbon metabolism by influencing photosynthesis and light signaling remains elusive, and investigations covering physiological processes, biochemical analysis, and organ-specific transcriptomes are still rare. In this study, physiological growth and biochemical indicators of grape plantlets under different qualities of monochromatic light (blue, red, and green) were determined to evaluate plantlet growth performance, and whole-genome transcriptome sequencing was used to provide a comprehensive identification of genes induced in response to different light quality treatments in leaves, stems, and roots. Finally, correlation network analysis (between differential genes and key physiological traits) was performed to screen candidate genes related to carbon metabolism and light signaling under different light qualities. The purpose of this work is to provide a foundation for optimizing light recipes for grapevine growth and will be beneficial for boosting the speed of production of healthy and high-quality grapevine planting materials.

## Results

### Effects of light quality on growth traits and leaf optical properties of grapevine plantlets

**Figure 1:**
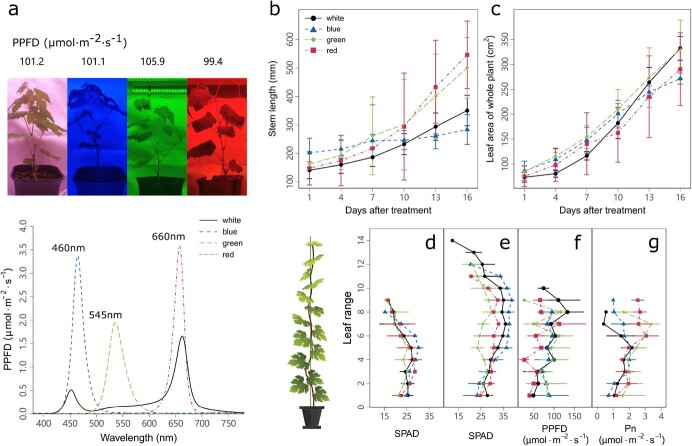
Comparisons of plantlet growth traits between different monochromatic light treatments. PPFD was measured 40 cm below the LEDs with a light spectrometer (LI-180, LI-COR) for the blue, green, and red monochromatic light treatments as well as the white light control, and the peak wavelength of each LED is indicated (**a**). Effect of light quality on plantlet stem length (**b**), whole plant leaf area (**c**), chlorophyll content (SPAD) at 5 days after treatment (**d**), SPAD at 20 days after treatment (**e**), *in situ* light intensity of each leaf within the canopy at 5 days after treatment (**f**), and *in situ* single leaf Np 5 days after treatment (**g**). Data are means ± standard errors (*n* = 3).

The growth traits were differentially affected by light qualities provided by monochrome LED lights (Fig. 1). Compared with white light, green and red monochromatic light increased stem length to a similar extent, while blue monochromatic light did not show evident effect (Fig. 1b). On the other hand, light quality did not lead to significant variation in single- or whole-plant leaf area (Fig. 1c and Supplementary Data Fig. S1). The spatial variation in leaf chlorophyll content (SPAD value) for each leaf in its *in situ* range in the plantlet showed a typical pattern, being lower for young and old leaves in the top and bottom ranges but being higher for functional adult leaves in the middle ranges (Fig. 1d and e). Light quality did not show a clear effect on the spatial variation in SPAD at 5 days after treatment (DAT) (Fig. 1d), but consistently impacted the SPAD at 20 DAT (Fig. 1e). At 20 DAT, green light reduced the SPAD of leaves in most ranges and red light reduced it to a lesser extent, while blue light increased the SPAD for leaves in the middle ranges of the canopy, in comparison with white light (Fig. 1e). Light quality exerted influences on the light intensity of the leaf blade at the *in situ* leaf position in a wavelength- and leaf-range-dependent manner (Fig. 1f). For the leaves of upper ranges, the three tested light qualities did not change leaf *in situ* light intensity (Fig. 1f). However, for the leaves in the bottom ranges, blue and green monochrome light increased leaf *in situ* light intensity by 46.63 and 37.09%, respectively, in comparison with their counterparts under white light, in agreement with the higher penetration capacity of blue and green light within the canopy (Fig. 1f). In addition, the Pn of leaves at their *in situ* position within the canopy showed significant differences between LED treatments in the upper leaf ranges, with the highest Pn under red light and lowest under white light (Fig. 1g). Moreover, single-leaf optical properties were quantified for light absorptance, reflectance, and transmittance from 400 to 720 nm (Supplementary Data Fig. S2). As expected, all the leaves showed lowest absorption and highest transmission for green light (500–570 nm) within the 400–650 nm scanning light wavelengths. Interestingly, the exact relative proportion of green light absorption by leaves varied with leaf range and the light quality under which the leaf grew. Particularly, the leaves under blue light showed the most evident increases in proportions of green light absorption from range 1 to range 11 (Supplementary Data Fig. S2) in comparison with leaves grown under white, green, or red light. In addition, the proportions of green light absorption were largely reduced in leaves grown under green light in comparison with those grown under white light (Supplementary Data Fig. S2).

### Effects of light quality on leaf gas exchange

**Figure 2:**
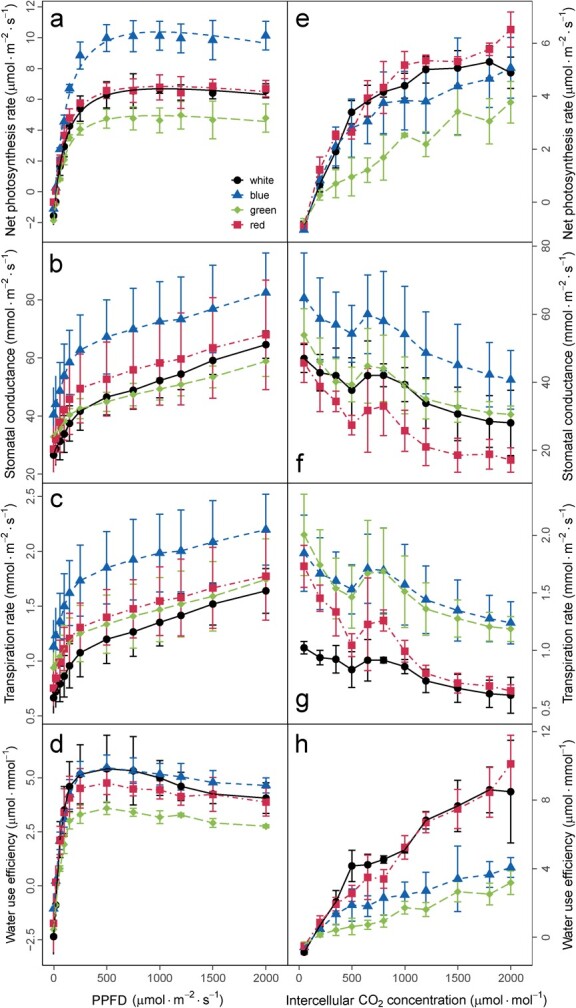
Response curves of leaf gas exchange to light (**a**–**d**) and CO_2_ (**e**–**h**) under different monochromatic lights with white light as control. Responses of net photosynthesis rate (**a**, **e**), stomatal conductance (**b**, **f**), transpiration rate (**c**, **g**), and water use efficiency (**d**, **h**) to light intensity (**a**–**d**) and intercellular CO_2_ concentration (**e**–**h**). Data are means ± standard errors (n = 3).

Light response curves of leaf gas exchange were produced for grapevine plantlets grown under the four monochromatic lights (Fig. 2a–d). For this, a just fully expanded and functional leaf that had emerged after the treatment (range 7) was enclosed in the leaf chamber of the photosynthetic machine (LI6400XT); a series of light intensities from the light source of the LI6400XT was supplied and the key parameters of leaf gas exchange were recorded. By doing so, leaves from different monochromatic lights were measured under the same environmental configurations including light quality, in order to assess the functional modifications in the leaf gas exchange systems induced by the different monochromatic lights.

Regardless of the light quality treatments, the Pn–PPFD (photosynthetic photon flux density) and Pn–Ci (intercellular CO_2_ concentration) response curves all showed the typical patterns, with Pn increasing as light intensity or Ci increased and finally stabilizing at a plateau at light or Ci saturation (Fig. 2a and e). However, the characteristic parameters of these response curves, such the as light compensation point, light-saturated photosynthesis (Pn_max_), and Ci-saturated photosynthesis, were differentially affected by the light qualities. In detail, blue light increased Pn_max_ by 39.58% in comparison with white light (Fig. 2a and Supplementary Data Fig. S3a), and this promotive effect on Pn was evident once PPFD was >150 μmol m^−2^ s^−1^ (Supplementary Data Fig. S4a) and became highly significant at PPFD >500 μmol m^−2^ s^−1^ (Fig. 2a). These results suggested that the effect of monochromatic blue light on Pn could vary as a function of light intensity. In alignment with the increase in Pn by blue light, it also coordinately increased stomatal conductance and transpiration rate and did not affect water use efficiency (WUE) in comparison with their counterparts under white light (Fig. 2b–d). In contrast, green light reduced Pn_max_ by 18.41% (Fig. 2a and Supplementary Data Fig. S3a). However, green light only slightly reduced stomatal conductance (Fig. 2b) and increased transpiration rate (Fig. 2c), leading to a significantly reduced WUE (Fig. 2d). On the other hand, red light did not show clear effects on Pn (Fig. 2a), but slightly increased stomatal conductance (Fig. 2b) and transpiration rate (Fig. 2c), leading to a marginal decrease in WUE (Fig. 2d). The light compensation point (LCP) was not affected by light quality (Supplementary Data Fig. S3b). By comparing the four traits investigated in the light response curves (Fig. 2a–d), WUE was more resilient to variations in light quality, as a result of counterbalance between Pn and transpiration rate (Fig. 2d).

The Pn–Ci curve established for leaves grown under distinct monochromatic lights demonstrated that the Pn of leaves grown under green light was maintained at a lower level in comparison with those under red, blue, and white light for a given Ci of >200 μmol mol^−1^ (Fig. 2e). In addition, the stomatal conductance of leaves under blue light was held at the highest level and that of red light at the lowest level when compared with those under other lights (Fig. 2f). With an elevation in Ci, the leaf transpiration rate diverged under different LED treatments, exhibiting a higher level under green and blue light in comparison with white and red light (Fig. 2g). Leaf WUE showed the largest extents of reduction (60.16–76.05%) under blue and green light, as the decrease in Pn and increase in transpiration rate under these lights will both decrease the WUE (Fig. 2h).

### Effects of light quality on distributions of dry mass, soluble sugars, and starch

**Figure 3:**
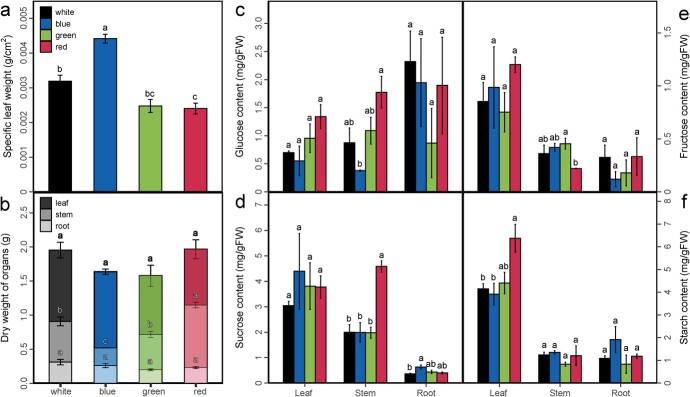
Dry mass, soluble sugars, and starch of grapevine plantlet organs under different monochromatic lights with white light as control. Specific leaf weight (**a**), dry weight of different organs (**b**), content of glucose (**c**), sucrose (**d**), fructose (**e**), and starch (**f**) in grapevine plantlet leaf, stem, and root. Data are means ± standard errors (*n* = 3). Bars labeled with different lowercase letters are significantly different at *P* < .05 level according to Duncan’s multiple range test.

Different light qualities affected the distribution of dry matter and the contents of major carbohydrates in above- and underground organs (Fig. 3). Compared with white light, specific leaf weight was increased significantly by 38.29% under blue light, and decreased significantly by 24.72% under red light. Green light reduced specific leaf weight by 22.38%, although the results were not significant in comparison with white light (Fig. 3a). Whole plantlets were dissected into three compartments – leaves, stem, and roots – and their dry masses were separately determined, but the dry weights of leaves and roots were not significantly influenced by light quality (Fig. 3b). However, the dry weight of the stem was significantly reduced (−57.07%) by blue light and increased (+53.60%) by red light, while it was not impacted by green light in comparison with white light (Fig. 3b). Soluble sugars and starch were quantified in leaf, stem, and root of grapevine plantlets grown under different light qualities (Fig. 3c–f). In comparison with white light, red light increased starch content by 53.63% in leaves and decreased fructose content by 51.36% in stems (Fig. 3e and f); blue light increased sucrose content by 71.95% in roots and decreased glucose content by 56.51% in stems (Fig. 3c and d); in contrast, green light did not show any significant effect on these traits (Fig. 3c–f).

### Differentially expressed genes induced by different light qualities

**Figure 4:**
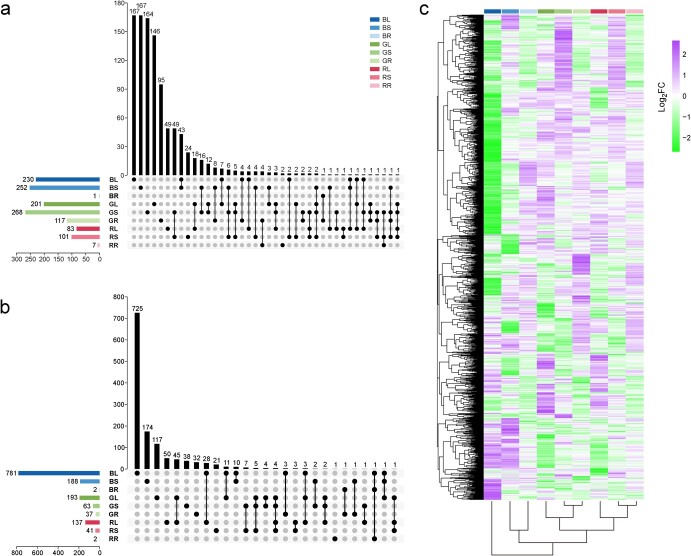
UpSet Venn diagram and expression pattern clustering analysis of DEGs in three organs under different monochromatic lights with white light as control. UpSet Venn diagram of upregulated (**a**) and downregulated DEGs (**b**), and expression pattern clustering analysis of DEGs (**c**). The color scale in (**c**) indicates the fold change in differential expression. B, blue light; R, red light; G, green light; W, white light; L, leaf; S, stem; R, root.

Whole-genome transcriptome was analyzed for leaves (L), stems (S), and roots (R) from the four light quality treatments (W, white; B, blue; G, green; R, red). Differentially expressed genes (DEGs) were obtained by comparing each organ of the grapevine plantlets under blue, green, and red light with that grown under white light, and this resulted in nine comparison groups (BL vs WL, GL vs WL, RL vs WL, BS vs WS, GS vs WS, RS vs WS, BR vs WR, GR vs WR, RR vs WR) (Fig. 4). A total of 2164 DEGs were identified among the nine comparison groups (Supplementary Data Table S1). There were overall more DEGs in the leaf and stem comparison groups than in the root comparison group (Fig. 4a and b). Moreover, there were more upregulated genes (Fig. 4a) than downregulated genes (Fig. 4b) in all comparison groups except for leaves under blue and red light, which induced more downregulated genes in leaves than upregulated genes. In the root comparison group (Fig. 4a and b), blue and red light barely induced DEGs (1–7 genes), while green light induced a higher number DEGs, with more upregulated DEGs (117 genes) than downregulated DEGs (37 genes). In addition, clustering analysis revealed that gene expression patterns of stem and root were more similar under monochromatic light, and gene expression profiles of different organs under blue light were even greater than those under red and green lights. Furthermore, the DEGs were mainly divided into four clusters. DEGs in cluster 1 were mainly upregulated in stem under green light and downregulated in leaf under blue light; DEGs in cluster 2 were downregulated in leaf under blue light and upregulated under green and red lights; DEGs in cluster 3 were mainly upregulated in root; DEGs in cluster 4 were mainly upregulated in leaf and stem under blue light and insensitive to the other two lights. (Fig. 4c).

### Gene Ontology enrichments induced by different light qualities

Gene Ontology (GO) enrichment analysis was conducted for up- and downregulated DEGs in leaves and stems under blue, green, or red monochromatic light in comparison with their counterparts under white light (Supplementary Data Figs S5 and S6). Results showed that the three monochromatic lights caused a handful of commonly enriched GO terms and a large quantity of light-specific GO terms (Supplementary Data Figs S5 and S6).

For the upregulated DEGs in leaves (Supplementary Data Fig. S5a), the three monochromatic lights induced common enrichments in monosaccharide, disaccharide, glucan, and starch metabolic processes, as well as oxidoreductase activity; blue and green light both caused enrichments in responses to sucrose, flavonoid biosynthesis, and cellular hormone metabolic processes; blue and red both caused enrichments in carbohydrate metabolic and iron ion binding processes; green and red light both caused enrichments in primary carbohydrate metabolism, secondary metabolism, and ion transport. On the other hand, blue light caused specific enrichments in photosynthesis, chlorophyll synthesis, light response, and primary carbohydrate and pigment metabolism; green light caused specific enrichments mainly in responses to sugars and lights; red light caused specific enrichments only in UDP-glucose metabolism (Supplementary Data Fig. S5a).

Among the DEGs downregulated in leaves (Supplementary Data Fig. S6a), the processes enriched in all three light quality treatments were mainly related to secondary metabolism and oxidoreductase activity, while genes related to single-organism biosynthetic process, oxidation–reduction process, photosynthesis, flavonoid synthesis process, chlorophyll synthesis, and light response were commonly enriched in green and red light-treated leaves. Moreover, the downregulated genes in blue light-treated leaves showed light-quality-specific enrichments in cellular metabolic process, primary metabolic process, response to hormone, histone H3–H9 methylation, sucrose metabolic process, etc. Green light caused specific enrichments in responses to metabolic process and single-organism metabolism for the downregulated genes (Supplementary Data Fig. S6a).

Among the DEGs upregulated in stems (Supplementary Data Fig. S5b), DEGs induced by the three lights were commonly enriched in processes related to high light intensity response and glycosyltransferase activity. Blue and green light both caused enrichments in responses to single-organism metabolic process, organic cyclic compound biosynthesis, and phenylpropanoid metabolic process. In addition, blue light caused specific enrichments in chloroplast, plastid, iron ion activity, and oxidation–reduction process; green light specifically caused enrichments in catalytic activity, cellular biosynthetic process transferase activity, and aromatic compound synthesis.

Among the DEGs downregulated in stems (Supplementary Data Fig. S6b), the three light treatments caused common enrichments in oxidation–reduction process and oxidoreductase activity. Blue and green lights both caused enrichments in responses to single-organism metabolic process, starch metabolism (carbohydrates), and monosaccharide metabolism (carbohydrates). Green and red light both caused enrichments in pigment and flavonoid metabolic processes. Moreover, blue light caused specific enrichments in carbohydrate metabolic processes, glucan metabolic processes, lignin metabolic processes, cell wall biogenesis, phloem, or xylem histogenesis etc. Green light caused specific enrichments mainly in isoprenoid and terpenoid metabolic processes, while red light caused specific enrichment only in regulation of biosynthetic process in the downregulated DEGs in stems (Supplementary Data Fig. S6b).

For the root transcript profiles, gene expression in root was responsive to green light while only a few DEGs were found under blue (up, 1; down, 2) and red light (up, 7; down, 2). Results showed the upregulated DEGs in root under blue light were enriched in photosynthetic membrane, PSII, thylakoid membrane, and photosystem process (Supplementary Data Fig. S7a). The downregulated DEGs under green light were mainly involved in monocarboxylic acid binding, carboxylic acid binding, organic acid binding, growth factor binding, signaling receptor activator activity, etc. (Supplementary Data Fig. S7b).

### Differentially expressed genes related to photosynthetic and central carbon metabolism processes

**Figure 5:**
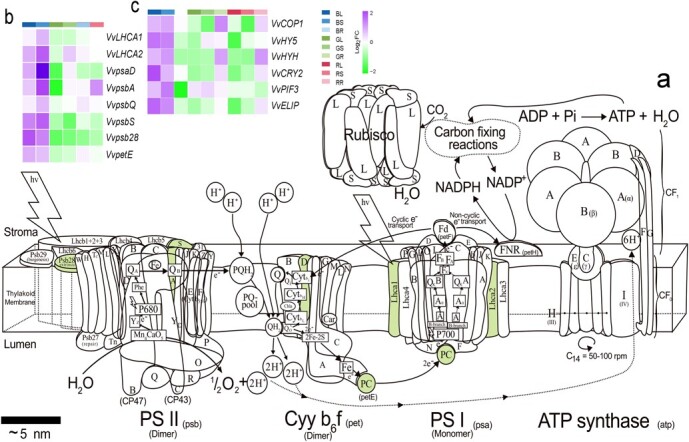
DEGs for photosynthetic structure and light signaling pathway. The structure of photosynthetic system is illustrated (modified from Allen *et al*., 2011 [32]) and the affected components are highlighted in green (**a**). Effect of light quality on the differential expression (fold change) of photosynthetic structure-related genes (**b**) and light signaling pathway genes (**c**). The color scale indicates the fold changes in comparison with white light. B, blue light; R, red light; G, green light; W, white light; L, leaf; S, stem; R, root.

GO analysis showed that the processes related to primary carbohydrate metabolism and photosynthesis were profoundly affected by the three monochromatic lights in comparison with white light, and therefore these genes were explored in more detail (Supplementary Data Table S1 and Supplementary Data Fig. S5). Twenty-four genes were affected by different light qualities involved in the photosynthetic system (Fig. 5a and b), light signaling (Fig. 5c), and central carbon metabolism pathways (Supplementary Data Fig. S8a). Photosynthetic system structure-related genes *VvLHCA1*, *VvLHCA2*, *VvpsaD*, *VvpsbA*, *VvpsbS*, *Vvpsb28*, and *VvpsbE* were consistently upregulated in blue light-treated leaves and stems and downregulated in green light-treated leaves, but showed no significant change under red light treatment (Fig. 5b). Light-signaling-related genes all showed coordinated responses to light quality between leaf and stem, while root responded mostly in a pattern opposite to leaf and stem (Fig. 5c). The genes encoding key players of light signaling, including *VvCOP1*, *VvHY5*, *VvHYH*, and *VvPIF3*, were significantly upregulated in leaf and stem under blue light treatment, but were downregulated in leaf and stem under green and red light. The blue light receptor-encoding gene *VvCRY2* was also upregulated in leaf and stem by blue light, while it was downregulated by green and red light (Fig. 5c). It is worth noting that *VvCOP1*, *VvHYH*, and *VvCRY2* were upregulated by green and red light in the root, in contrast to their responses in leaf and stem (Fig. 5c).

In the central carbon metabolic pathway, multiple genes were responsive to different light qualities (Supplementary Data Fig. S8). *VvAMY1*, encoding amylase, which catalyzes the hydrolysis of starch, was downregulated in the stems and upregulated in the roots by all the three monochromatic lights in comparison with white light, while it was not significantly affected by light quality in leaf (Supplementary Data Fig. S8a); *VvPK*, encoding pyruvate kinase, was upregulated in the leaf and stem by all the three monochromatic lights (Supplementary Data Fig. S8a); *VvSPS*, encoding sucrose phosphate synthase, was upregulated in the leaves by blue light and downregulated by green light (Supplementary Data Fig. S8a); *VvSUS*, encoding sucrose synthase, was downregulated in leaf and stem under blue light (Supplementary Data Fig. S8a); *VvALDA1*, encoding fructose-bisphosphate aldolase (class I), was upregulated in the leaf and stem under blue light, upregulated in the root, and downregulated in the leaf under green light (Supplementary Data Fig. S8a); the hexose transporter encoding gene *VvHT5* was significantly downregulated in the stem and root by all the three monochromatic lights (Supplementary Data Fig. S8b); the sugar transporter encoding genes *VvSWEET3* and *VvSWEET4* were upregulated in the root under green and red light (Supplementary Data Fig. S8b).

### Correlation network between differentially expressed genes and physiological traits

**Figure 6:**
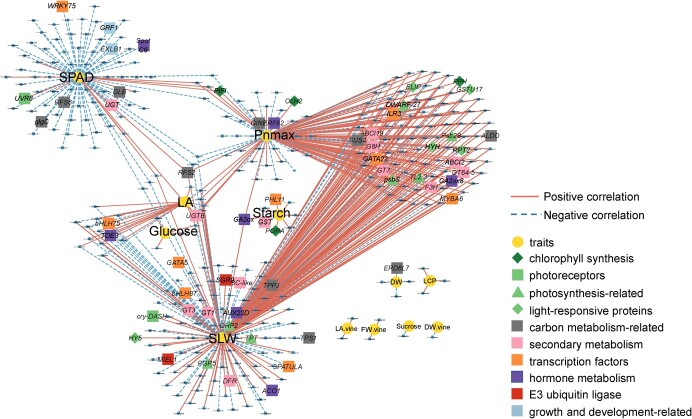
Correlation network between physiological and biochemical traits and DEGs in leaves grown under different light qualities. SPAD represents the estimation of chlorophyll content, Pn_max_ represents light-saturated photosynthesis rate, and Glucose, Sucrose, and Starch represent their corresponding concentrations. LA, single leaf area; SLW, specific leaf weight; DW, leaf dry weight; LCP, light compensation point of leaf photosynthesis. All these traits were measured for the range 7 leaf (see Fig. 1). LA.vine, FW.vine, and DW.vine denote leaf area, fresh weight, and dry weight of the whole plant, respectively. Physiological and biochemical traits and genes are represented with different colors and symbols, and genes related to photosynthesis, light signaling, and transcription factors are highlighted with larger symbols.

The Pearson correlations between physiological traits and DEGs were analyzed by pooling results from vines grown under white, blue, green, and red light for each organ. A total of 493 correlated gene–trait pairs (*P*-value ≤.001, |*r*| > 0.8) were screened in leaves, which involved a total of 14 traits and 358 genes, with 302 positively and 191 negatively correlated pairs (Fig. 6, Supplementary Data Table S2). The correlation network was divided into four modules and several isolated satellites. The first module was mainly related to the light-saturated photosynthetic rate (Pn_max_), which was significantly correlated with two genes (*VvPIFI*, *VvCLH2*) related to chlorophyll synthesis, one gene (*VvGIN2*) related to carbon metabolism, and one gene (*VvERF62*) related to the hormone metabolic pathway (Fig. 6). The second module was mainly related to specific leaf weight (SLW), and the correlated genes included photosynthesis-related genes (*VvPGR5*, *VvOHP2*, *VvTPT*), carbon metabolism-related genes (*VvTPPJ*, *VvTPS1*), transcription factors, genes encoding E3 ubiquitin ligases (*VvSGR9*, *VvMIEL1*), and a gene encoding photoreceptors (*Vvcry-DASH*). The genes in the third module were associated with both Pn_max_ and SLW, suggesting that these two traits might be co-regulated by the genes in this module (Fig. 6). Interestingly, genes in this module were mainly involved in photosynthesis, carbon metabolism, secondary metabolism, and hormone metabolism, suggesting that there might be a tight coordination between photosynthesis and carbon metabolism in response to different light qualities. Particularly, the relative expressions of photosynthesis system structure genes (*VvpsbS* and *Vvpsb28*), light signaling genes *VvHYH* and *VvELIP* (early light-induced protein), and sugar metabolism genes (*VvSUS4* and *VvALDA1)* were significantly and simultaneously associated with Pn_max_ and SLW (Supplementary Data Fig. S9). Moreover, these genes overlapped with the genes screened based on GO enrichments (Supplementary Data Fig. S8), suggesting that they may play an important role in response to light quality and in regulating plant Pn_max_ and carbon metabolism.

Similarly, the correlation networks between physiological traits and DEGs in stem and root were also determined to investigate the key genes responsible for the growth of stem and root under different monochromatic lights (Supplementary Data Fig. S10). In the stem network, a total of 180 correlated gene–trait pairs with strong correlation (*P* ≤ .002, |*r*| > 0.8) were screened, and a total of 9 traits and 126 genes were associated, with 105 positively and 75 negatively correlated pairs (Supplementary Data Fig. S10a). The genes correlated with stem growth and sugar content traits were mainly involved in cellulose metabolism, carbon metabolism, and E3 ubiquitin ligase, including the photoreceptor *Vvcry*-*DASH*, the sugar transporter *VvSWEET3,* the transcription factors *VvWRKY14* and *VvMYBR1*, and carbon-metabolism-related genes *VvGOLS1* (galactinol synthase 1) and *VvGAPD* (glyceraldehyde-3P-dehydrogenase). For the root network (Supplementary Data Fig. S10a), there were 20 positive and 9 negative correlated gene–trait pairs, including 26 genes and five growth traits. Among them, the light signaling gene *VvELIP* (early light-induced protein) was the only gene that responded to light quality systematically from leaf to root, and the other correlated genes were specifically identified in roots and dominantly involved in carbon metabolism and secondary metabolism (Supplementary Data Fig. S10a).

## Discussion

### Effects of light quality on plant source and sink organs

Light is crucial for plant living, and the perception of light dictates plant growth, morphology, and developmental changes [33]. Light is also the driving force behind photosynthesis, and light intensity and spectral composition act as signaling factors during plant development [34]. Previous research in grapevine mainly focused on fruit secondary metabolism in response to different monochromatic lights, and revealed that anthocyanins and other polyphenolic compounds were sensitive to both the light intensity and light quality provided by LEDs [5, 35]. However, few studies have investigated plant growth, photosynthetic performance, and carbon allocation in the plant through the integration of transcriptomes and phenotypes of different organs. In the present study, strict isolation of external light sources and elaborated control of chamber conditions provided a solid base for grape plantlet growth and transcriptional profiling under different monochromatic lights.

LED lights showed distinct effects on the growth performance of grapevine plantlets. Specifically, blue monochromatic light strengthened the growth of plantlet leaf and root (increased leaf SLW and starch content of root; Fig. 3a and f) and reduced stem quality (decreased stem length and glucose/fructose content of stem; Figs 1b and 3c and e). On the other hand, red and green monochromatic light enhanced leaf Pn (Fig. 1e) and stem growth (increased stem length; Fig. 1b) while leaf SLW and SPAD were decreased (Fig. 3a). Similar results have been found in *C. annuum* [20], tomato [36], poinsettia [37], and grape (*in vitro*) [29]. The phenomenon of the enhancement of stem elongation and reduced carbon investment in other organs was similar to the known shade-avoidance syndrome [29]. The occurrence of the shade-avoidance syndrome was not only mediated by the low red:far red (R:FR) ratio, but can also be induced by other signals, including red light, blue light, ultraviolet (UV) radiation, and the reflection and transmission of green light [38]. Additionally, to investigate whether the variation in plant growth under different light qualities was due to the selective absorption of light wavelengths by leaves, the light intensity and optical properties of the *in situ* leaf position were determined (Fig. 1f and Supplementary Data Fig. S2). Results showed that the absorption of red light and blue light by the leaves was greater than that under green light, in line with previous studies [39].

Dissecting the source–sink status under LED lights is of importance for high-quality plantlets. In the source side, increasing photosynthesis was a powerful strategy to improve source strength [40], and our results showed that blue light increased leaf SPAD, SLW, and photosynthetic potential (Figs 1e and 3a and Supplementary Data Fig. S3). Red light enhanced the starch content of leaves (Figs 3d and f), while green light did not promote the source organs. Moreover, it is known that both the source and sink organs co-limited the plant carbon flux, and enhancement only in the leaf may lead to sink limitation [41]. For the sink side, sucrose content was increased under blue light only in roots, and enhanced by red light in stems (Fig. 3d). This suggested that the photoassimilates from leaves would be provided to the sink according to its resource demand rather than flowing to source organs equally. A similar study showed that red, blue, and green light increased the soluble sugar content in grape plantlet leaf *in vitro*, while red and green light also increased leaf starch content [29]. This revealed that the carbon flow between different organs of a pot-grown plantlet differed from that of a tender plantlet *in vitro*, and the growth response of a plantlet in the actual light environment has more reference significance for the construction of a highly efficient vine nursery. Moreover, we noticed that the biological variabilities in root glucose content, as well as other traits, were larger than those of other tissues. These higher variabilities in roots may partially be attributed to their complex hierarchical branching, with different types of roots. Although we collected only the white lateral roots, more biological replicates might be required to further improve stability and statistical reliability for roots in future studies. It is also worth noting that green light decreased the leaf photosynthetic potential and had no effect on carbon flow regulation, which highlighted the central role of photosynthesis in regulating sink–source relationships.

Monochromatic light had different effects on DEGs corresponding to central carbon metabolism and sugar transporters (Supplementary Data Fig. S8). The genes involved in central carbon metabolism (*VvAMY2*, *VvSPS*, *VvALDA1*, and *VvPK*) were upregulated in plantlets under blue light, which could be partly attributed to the increased leaf SLW. Moreover, the upregulated *VvPK* in leaf and stem under red light was also associated with the increases in starch and sucrose contents in leaf and stem. As the main photoassimilate transported from source (leaf) to sink (such as root) through the phloem system, sucrose is essential for root growth [42]. In this study, the sugar transporter genes *VvSWEET3* and *VvSWEET4* were upregulated in roots under red and blue light, indicating that they might be regulated by long-distance signals. It is worth noting that the expression patterns of these genes were basically the same in leaf and stem, but different in root, suggesting that the expression of genes related to carbon metabolism in above-ground parts was mainly affected by monochromic light, while that in root could be mainly dependent on long-distance signals from shoot to root [31].

### Response of leaf photosynthesis and plant carbon assimilation to light quality

The light response curve and Pn–Ci curve are important ways to explore the photosynthetic physiological and ecological adaptions of plants under environmental changes. In our study, the Ci–Pn curve was obtained under different light qualities at a relatively low light intensity (~101 ± 5 μmol m^−2^ s^−1^) and it showed that the photosynthetic rate of leaves did not show clear differences under the lower range of CO_2_ concentration among the different light qualities (Fig. 2e). However, under higher CO_2_ concentration ranges (250–1200 μmol mol^−1^), Pn was gradually decreased by blue light in comparison with white light (Fig. 2e). This indicated that the effect of blue light on Pn was dependent on the light intensity and CO_2_ concentration, and its effect may even reverse under different combinations of light intensity and CO_2_. This may explain the discrepancies reported in the literature about the effects of blue light on Pn [13, 29, 33]. On the other hand, the PPFD–Pn curve also showed that a low fluence rate of blue light was sufficient to induce the increase in stomatal opening and Np (Supplementary Data Fig. S4a and b), which confirmed that the regulation of stomata by blue light was rapid and guard-cell-specific [43]. Transcriptome results showed that the expression of blue light photoreceptor *VvCRY2* could mediate stomata behavior under blue light. Moreover, a gene encoding a copper transport protein (*VIT_213s0067g02140*) was also increased under blue light, which could be responsible for the inflow of copper ions and further decrease water potential in the guard cell (Supplementary Data Table S1). Compared with blue and red light, green light is one of the least economical lighting strategies; Pn–PPFD/Ci curves showed that green light did not increase the Np but decreased the WUE. Interestingly, stomatal conductance was insensitive to green light, which indicated that the increased transpiration rate should not be attributed to *g*_s_, but to mesophyll conductance (*g*_m_). A study also showed that tomato (*Solanum lycopersicum* L. cv. ‘Microtom’) leaf mesophyll conductance was higher under RGB (red–green–blue) light when compared with that under RB (red–blue) light [44]. These results emphasized the importance of mesophyll conductance in the response to green monochromatic light, and the underlying mechanisms warrant further investigations.

### Transcriptional profile in different organs and mining key genes responsible for light quality

LED monochromatic lights induced whole-transcriptome reprograming in plantlet leaf, stem, and root. GO annotation analysis showed that DEGs under different lights were uniformly enriched in light signal transduction, PSII composition, chloroplast structure, and carbon metabolism. Especially, there were overall more DEGs in leaf under blue light (up, 230; down, 781) compared with numbers under red and green light. The downregulated DEGs in blue light were uniquely enriched in GO term ‘response to hormone’, among which there were many genes associated with ethylene biosynthesis and signaling (*allene oxide cyclase*, *ERF014*, *ERF023*, *ERF026*, *RAP 2–7*, *ABR1*, *CRF4*), suggesting potential crosstalk between blue light and ethylene signaling. Similarly, a recently study in pear found that HY5, a key light signaling player, could bind to the promoter of ACC synthase 1, a key enzyme of ethylene biosynthesis, and consequently decrease ethylene production [45].

Roots anchor the plant in soils and are responsible for absorbing water and inorganic nutrients from below ground. Therefore, coordinated growth of shoot and root is essential for plants coping with changing environments. In this study, blue light induced three DEGs in root (up, 1; down, 2) and red light induced nine DEGs in root (up, 7; down, 2), while green light induced 154 DEGs in root (up, 117; down, 37). Some work has confirmed that light signals can be transmitted from the shoot to the root and affect root morphology and nutrient absorption. For example, HY5 is a central transcription factor regulating plant photomorphogenesis, and it can bind to LHCB13 [46] and LHCA4 [47] under light and temperature changes. Moreover, HY5 has also been shown to act as a shoot-to-root signal transducer in regulating nitrate absorption and lateral root emergence under blue light [48]. A study in *Arabidopsis* showed that some photoreceptors can inhibit COP1 and then regulate the expression of auxin transporters and coordinate growth between shoot and root [31]. In our experiment, expressions of the *VvHY5*, *VvCOP1*, *VvHYH*, *VvCRY2*, and *VvPIF3* genes were increased by blue light and inhibited by red/green light in leaf and stem, but a consistent trend has not been found in roots embedded in the soil (Fig. 5c). However, the gene expressions of *VvHYH* and *VvPIF3* were indeed decreased in root under blue light, *VvCOP1* was increased in root under green light, and *VvCOP1* and *VvHYH* were increased in root under red light, compared with that under white light. Moreover, compared with red and blue light, the green-light-mediated downregulation of many genes in the root implied that there may be unique light signal transduction at the global level for green light. Hence, Venn analysis was conducted to investigate the common genes both in leaf and root under green light (Supplementary Data Fig. S7c and d). Results showed that two genes coding uncharacterized proteins have been found. Furthermore, the Venn analysis also showed that there were seven genes shared between stem and root under green light (Supplementary Data Fig. S7c and d, Supplementary Data Table S1), including the transcription factor WRKY72, a BTB/POZ and TAZ domain-containing protein 1, and a subtilisin-like protease, SBT1.1. The BTB and TAZ domain protein has been reported as occupying an integral position in a complex and being integrated into a signaling network to regulate ABA- and sugar-mediated inhibition of germination [49]. WRKY72 was reported to regulate terpene biosynthesis by binding to the promoter of linalool synthase TPS5 [50]. Overall, these results suggested that green light uniquely triggers a global signaling reaction from shoot to root.

Correlation network and correlation analysis between DEGs and physiological traits were conducted to investigate the key genes responsible for different monochromatic lights treatments. Result showed the *VvpsbS*, *Vvpsb28*, *VvHYH*, *VvELIP*, *VvSUS4*, and *VvALDA* genes were correlated tightly with leaf Pn and leaf SLW. *VvpsbS* and *Vvpsb28* encode Psb proteins that are functional constituents of the PSII core complex [51]. *VvHYH*, the *HY5* homolog, could be related to the downstream UV-B signals and modulate lateral root development [52]. The early light-induced protein (ELIP) was originally thought to be a photoprotectant for reducing high light damage, and the promoter of *VvELIP* could be bound by HY5 and be involved in UV-B radiation and high light responses [53]. In this study, the expression of *VvELIP* was highly correlated with the fructose content of root (Supplementary Data Fig. S10b), revealing that *VvELIP* might be involved in shoot–root signaling. In general, these aforementioned six genes are most likely to play an important role in the regulation of photosynthesis and carbon metabolism under LED monochromatic lights and could be candidate genes for further study.

In summary, the effects of different monochromatic lights (white, green, blue, and red) on grapevine growth, leaf photosynthesis, whole-plant carbon allocation, and transcriptome reprograming were comprehensively investigated. Results showed that both blue and red lights were favorable for grape plantlet growth while green light reduced all photosynthetic indexes of the grape plantlet, compared with that under white light. PPFD/Ci–Pn curves indicated that the effect of blue light on Pn is dependent on light intensity and CO_2_ concentration and could be activated at a low fluence rate. Furthermore, RNA-seq analysis of different organs (leaf, stem, and root) indicated there was global light signaling conduction under light treatments and that *VvCOP1*, *VvHY5*, *VvHYH*, *VvPIF3*, and *VvELIP* were the key genes in the process of shoot-to-root signaling. Moreover, green light may have a unique global signaling mode compared with red and blue lights. Furthermore, the correlation network between DEGs and key traits indicated that *VvpsbS*, *Vvpsb28*, *VvHYH*, *VvSUS4*, and *VvALDA* were pertinent candidate genes in the plant in response to different light qualities. This work provides a foundation for optimizing the light recipe for grape plantlets and strengthens the understanding of light signaling and carbon metabolism under different LED monochromatic lights.

## Materials and methods

### Plant material and growth conditions

This study was conducted in a controlled indoor culture chamber. One-year-old virus-free plantlets of the grapevine cv. ‘Beihong’ (‘Muscat Hamburg’ × *Vitis amurensis*) were grown in a substrate of a mixture of perlite, vermiculite, and sandstone (1:3:3, v/v/v) under white light. After the emergence of the fifth leaf, plantlets were transferred to four light qualities provided by LEDs, including white light (W, the control group), blue light (B, peak at 460 nm), green light (G, peak at 545 nm), and red light (R, peak at 660 nm) with PPFD at 101 ± 5 μmol m^−2^ s^−1^ measured 40 cm below the LEDs with a light spectrometer (LI-180, LI-COR, USA) (Fig. 1a). Photoperiod, temperature, relative humidity, and CO_2_ concentration were 14 h, 26 ± 2°C, 65 ± 10% and 600 ± 30 μmol mol^−1^, respectively. Grapevine plantlets were attributed to the four light qualities with a complete random block design with three biological replicates and each replicate contained five plantlets with homogeneous vigor.

### Measurement of growth parameters and physiological traits

Each leaf was tagged with a range number, with 1 for the basal leaf and the largest number for the top leaf of the plantlets (Fig. 1). Single leaf area (LA) and stem internode length (SL) (*n* = 3) were monitored at intervals of 3 days from the first day after treatment (DAT). The LA of the whole plant was estimated non-destructively during growth by measuring leaf length and maximum width according to the method published by Montero *et al*. [54]. Different tissues (leaf, stem, and root) of grapevine plantlets were collected separately on the last day of the treatment (DAT = 25), and used to obtain fresh weight (FW), dry weight (DW), and sugar content. Specific leaf weight (SLW) was determined by the following formula: single leaf DW/single leaf area (confirmed by ImageJ).

A CI-710 leaf spectrometer (CID Bio-Science, Camas, WA, USA) was used to capture single-leaf optical properties (light absorption, reflectance, and transmittance from 400–720 nm). Three positions of each single leaf were measured to obtain a reliable mean value.

Gas exchanges and SPAD values (total chlorophyll content) of every leaf were measured. Not photosynthesis rate, stomatal conductance, transpiration rate (E), and water use efficiency (WUE, Pn/E) were measured by a portable photosynthesis system (LI-6400XT, LI-COR, USA) under the different light qualities, with 650 ± 10 μmol mol^−1^ CO_2_ concentration and 45 ± 10% relative humidity. The SPAD values were measured by using a chlorophyll meter (SPAD-502, Konica Minolta, Tokyo, Japan).

Light response curves and CO_2_ response curves (Pn–Ci curves) were extracted from leaves of range 7 using an LI-6400XT. With CO_2_ concentration at 650 ± 10 μmol mol^−1^ and relative humidity at 45 ± 10%, the measurement of light response curves was made after sufficient light induction, and the PPFD (μmol m^−2^ s^−1^) was set at the following levels: 2000, 1500, 1200, 1000, 750, 500, 250, 150, 100, 50, 20, and 0. The curves were fitted using the modified rectangular hyperbola model of Ye and Zhao [55], and Pn_max_ was estimated with the same method. To obtain Pn–Ci curves, leaves in the transparent leaf chamber were exposed to a CO_2_ concentration gradient of 650, 500, 350, 200, 50, 650, 650, 800, 1000, 1200, 1500, 1800, and 2000 μmol mol^−1^.

### Measurement of soluble sugars and starch

The concentrations of soluble sugars (glucose, fructose, and sucrose) and starch of plantlet leaf, stem, and root were determined enzymatically using an ELISA plate reader (Synergy HIMF, BioTek, USA), with a d-fructose/d-glucose assay kit and invertase from Megazyme (K-FRUGL and E-INVPD, Megazyme, Ireland). Starch content was measured using the colorimetric method [56] with a starch content assay kit (Cat #BC0700, Solarbio, China).

### Transcriptome analysis

Total RNAs of leaf, stem, and root under LED monochromatic lights were extracted using a Quick RNA isolation kit (Bioteke Corporation, Beijing, China). Transcriptome sequencing was accomplished by Biomarker Technologies Corporation (Beijing, China). Paired-end FASTQ files were trimmed for adapter and low-quality reads by using Trimmomatic v0.39 [57], and the read statistics were assessed by FastQC (http://www.bioinformatics.babraham.ac.uk/projects/fastqc/). The clean sequencing reads were mapped to the grape reference genome PN40024 12X v2.1 by STAR v.2.7.9a [58] and calculations of read counts and FPKM were executed by RSEM v1.3.1 [59]. By using DESeq2 v1.34.0 [60] in the R package, differential expression analysis was performed between treatments at the same organ level, and DEGs were screened out by the terms of padj <.05 and log_2_FC > 1. GO enrichment analysis and Kyoto Encyclopedia of Genes and Genomes (KEGG) pathway enrichment were conducted using AgriGO v2.0 [61] and ShinyGO v0.75 [62], respectively. The Pearson correlation coefficient between traits and FPKM of DEGs was determined by R software v4.0.5, and the networks were visualized using Cytoscape v3.9.0 [63]. Data visualization was carried out through R and TBtools v1.098691 [64]. The transcriptome data were deposited in the Grape-RNA database (http://www.grapeworld.org/gt/index.html).

### Statistical analysis

All experiments were performed with three biological replicates and each replicate contained five plantlets with homogeneous vigor. Significant differences at *P* < .05 level were determined according to Duncan’s multiple range test.

## Supplementary Material

Web_Material_uhad160

## Data Availability

The data underlying this article are available in the article and in its online supplementary material.
