# Supplementary material for: Grapevine plantlets respond to different monochromatic lights by tuning photosynthesis and carbon allocation
Source: Hortic Res. 2023 Aug 8;10(9):uhad160. doi: 10.1093/hr/uhad160 (PMC10500148; doi:10.1093/hr/uhad160)
Supplement: Web_Material_uhad160 [file web_material_uhad160.zip › Supplementary Figures.docx]

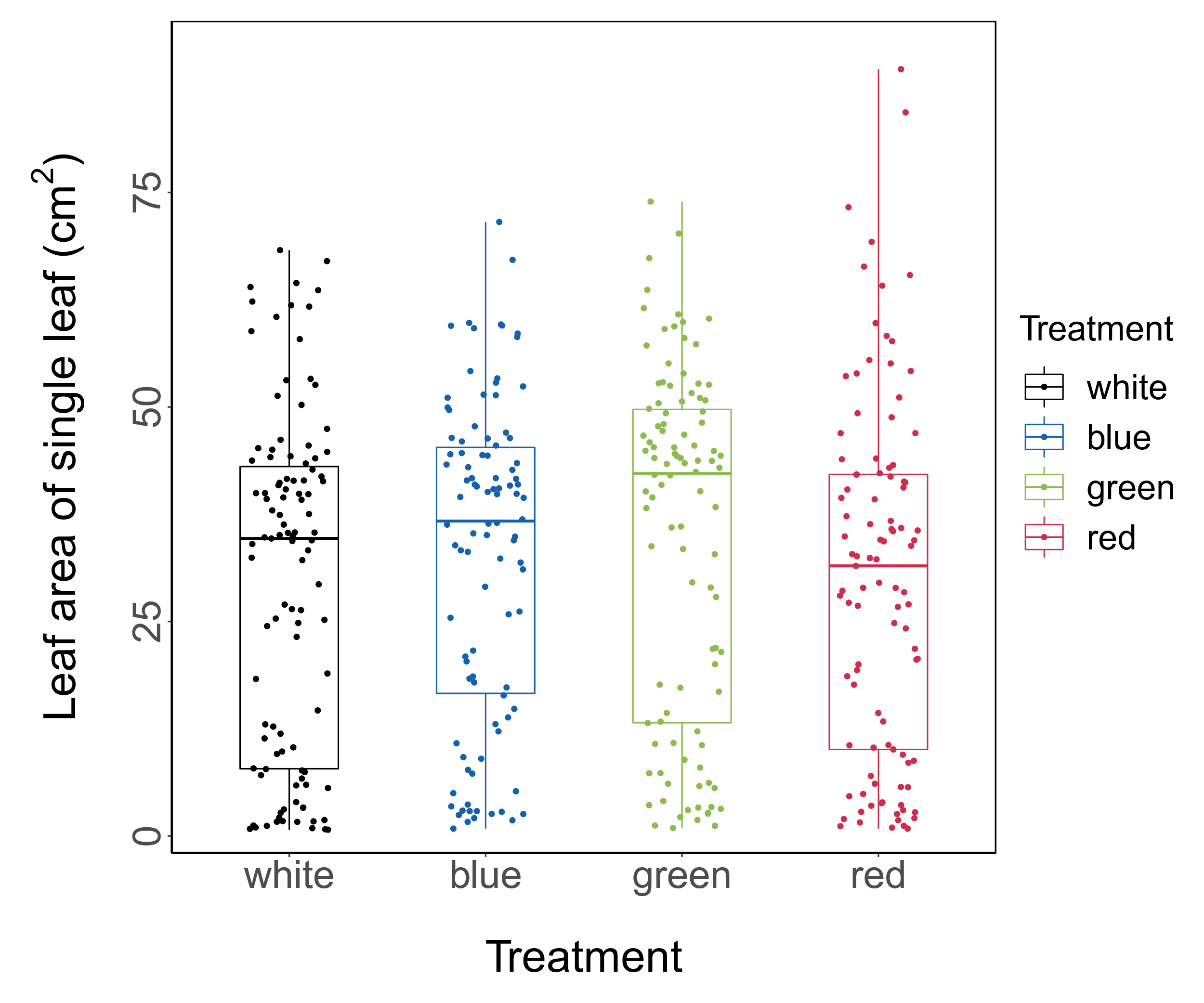


**Supplementary Fig. 1 Single leaf area of grape plantlets under different monochromatic lights.** white, white light; blue, blue light; green, green light; red, red light. Each dot represents a leaf area value of a single leaf. The box plot display medians (horizontal line) the 75th and 25th percentiles (top and bottom box) and the lower whiskers extend to data no more than 1.5× the interquartile range from the upper edge and lower edge of the box, respectively.


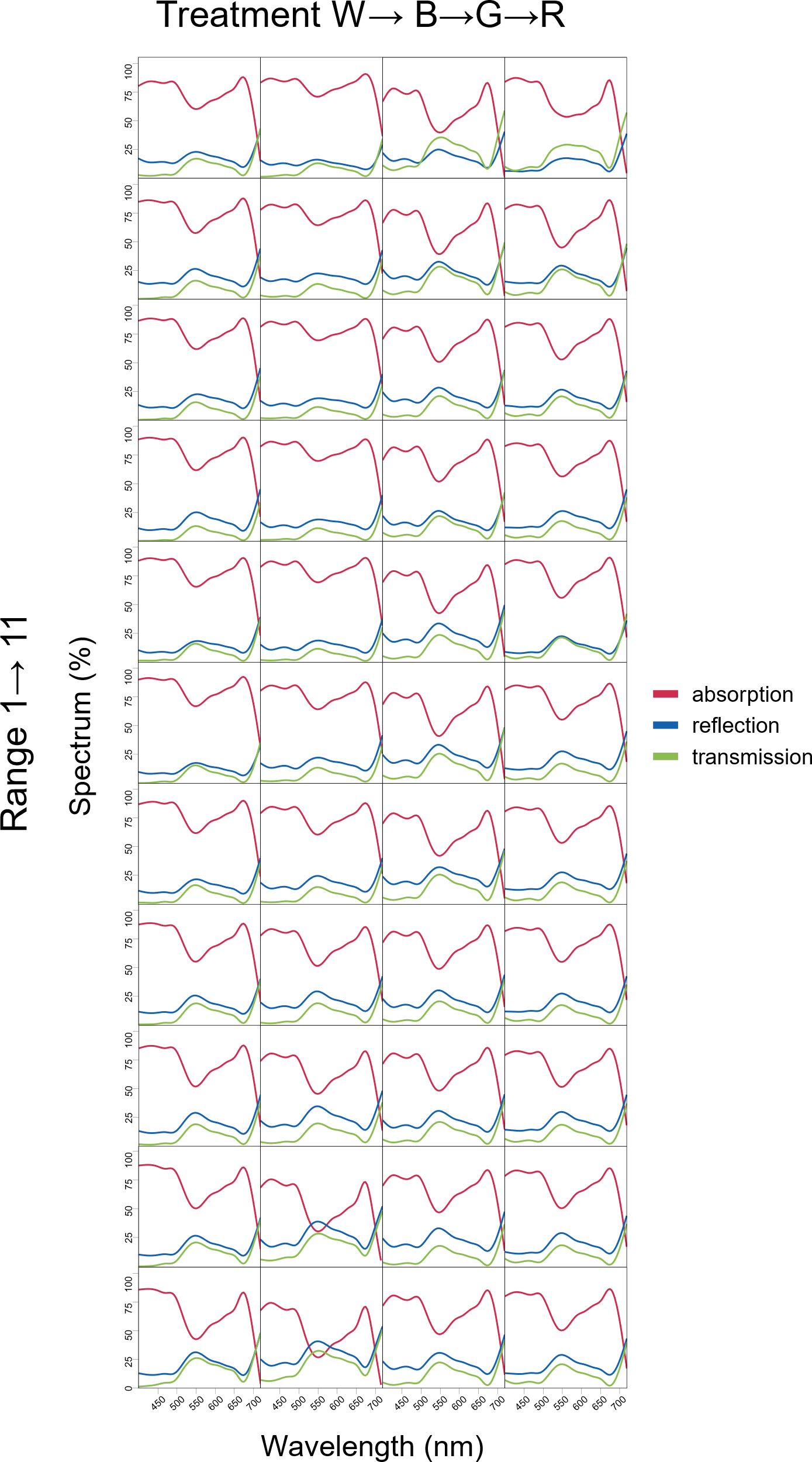


**Supplementary Fig. 2 *In-situ* leaf optical properties for each single leaf of range 1 (bottom) to 11 (top) under different monochromatic light treatments.** W, white light; B, blue light; G, green light; R, red light. For each column, the position of the figure from bottom to top represents the leaf range (1-11, see also Fig. 1).


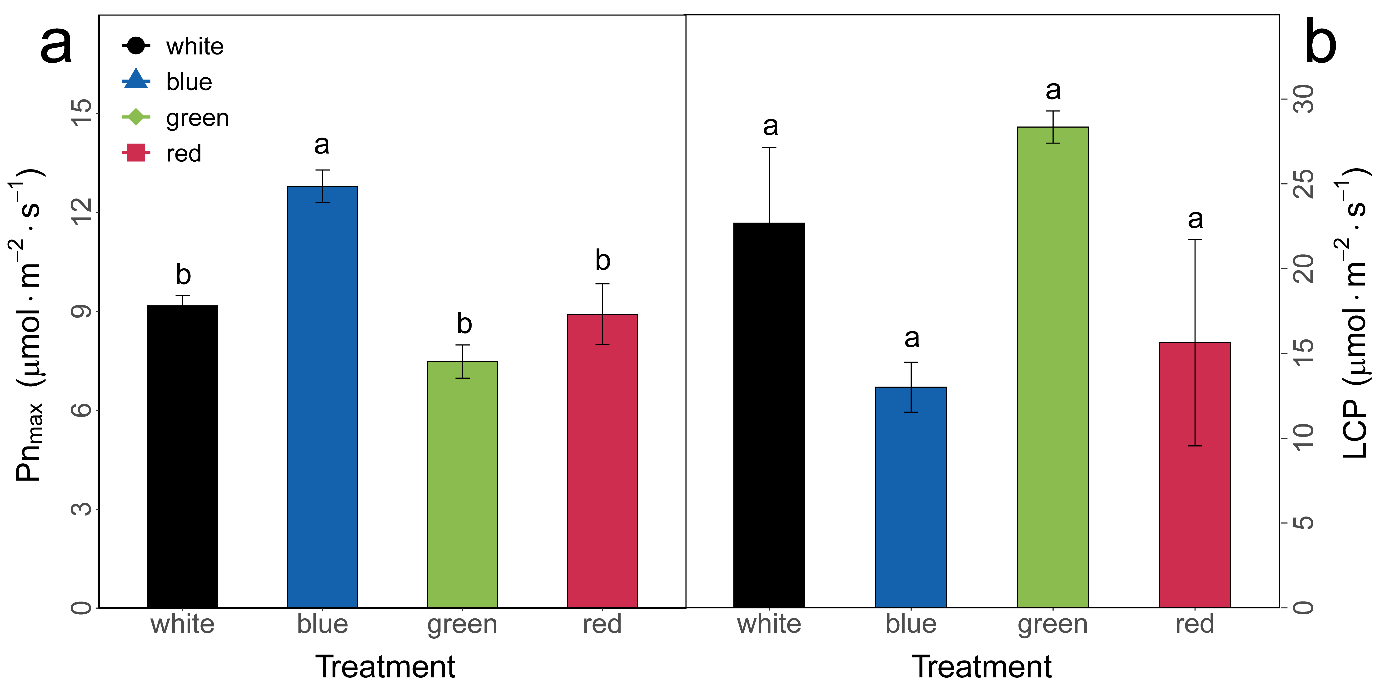


**Figure. S3 The light-saturated photosynthesis rate (Pn_max_)and light compensation point (LCP) under different monochromatic lights.** Data are means ± SEs (n=3). Different letters indicate significantly different at *P* < 0.05 level according to Duncan’s multiple range test.

**
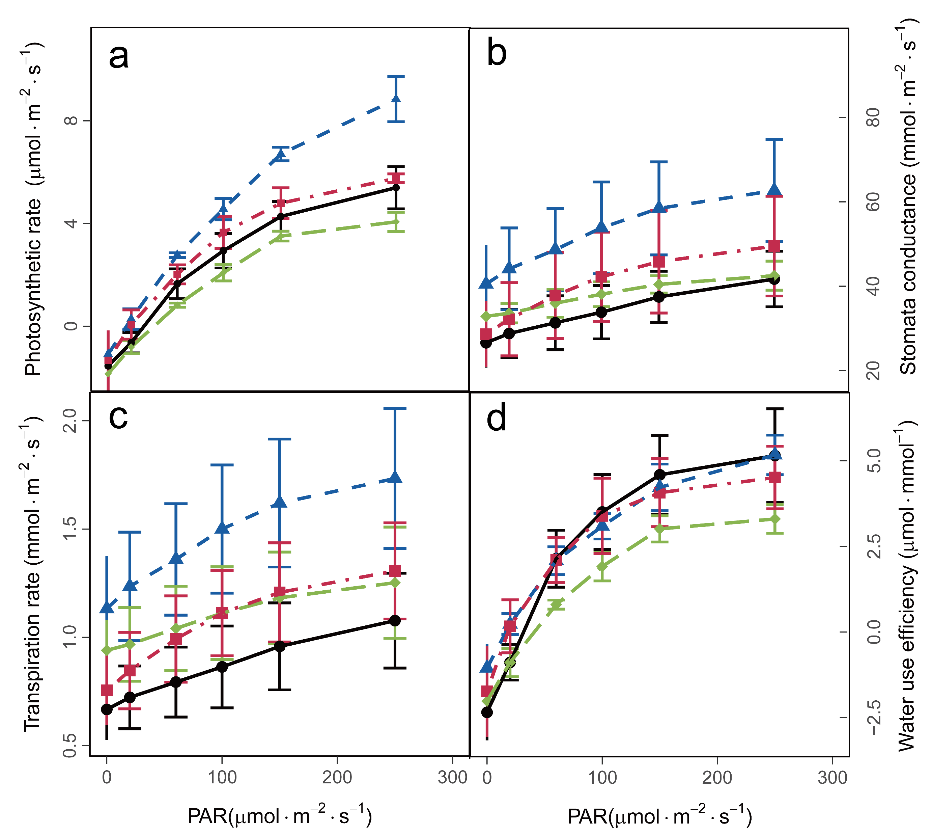
**

**Figure. S4 An enlarged view of leaf Pn-PPFD curve when the PAR was below 300 μmol·m^-2^·s^-1^ in response to different monochromatic lights.** Net photosynthesis rate (a), stomatal conductance (b), transpiration rate (c), water use efficiency (d). The color of dashed lines represents different monochromatic treatments. PAR, photosynthetic active radiation. Data are expressed as the means ± SEs (n=3).


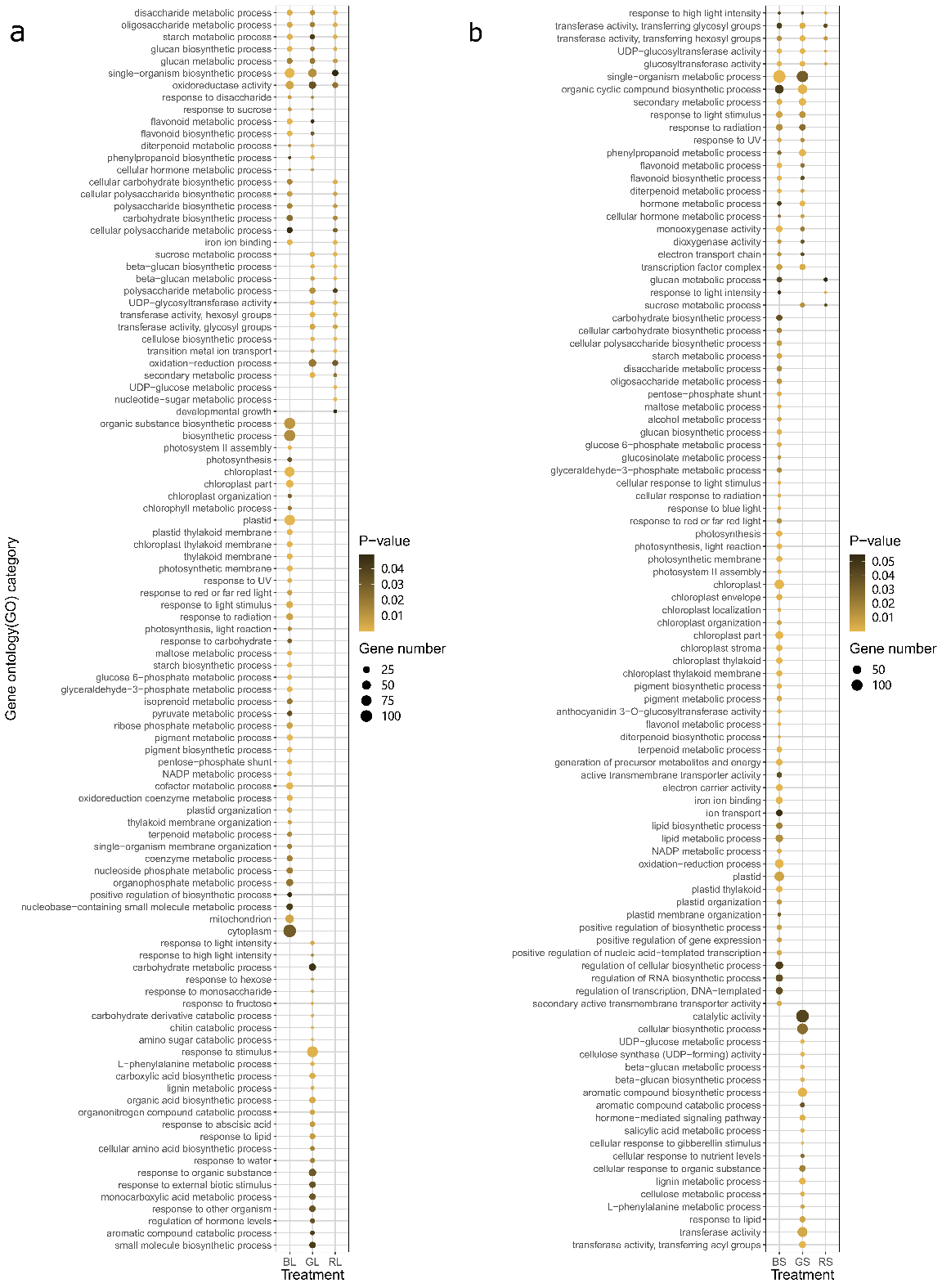


**Supplementary Fig. 5 GO functional enrichment of up-regulated in leaf (a) and stem (b) in response to monochromatic lights.** The color scale indicates the significance of enrichment and the circle size indicates the number of genes enriched in the term.


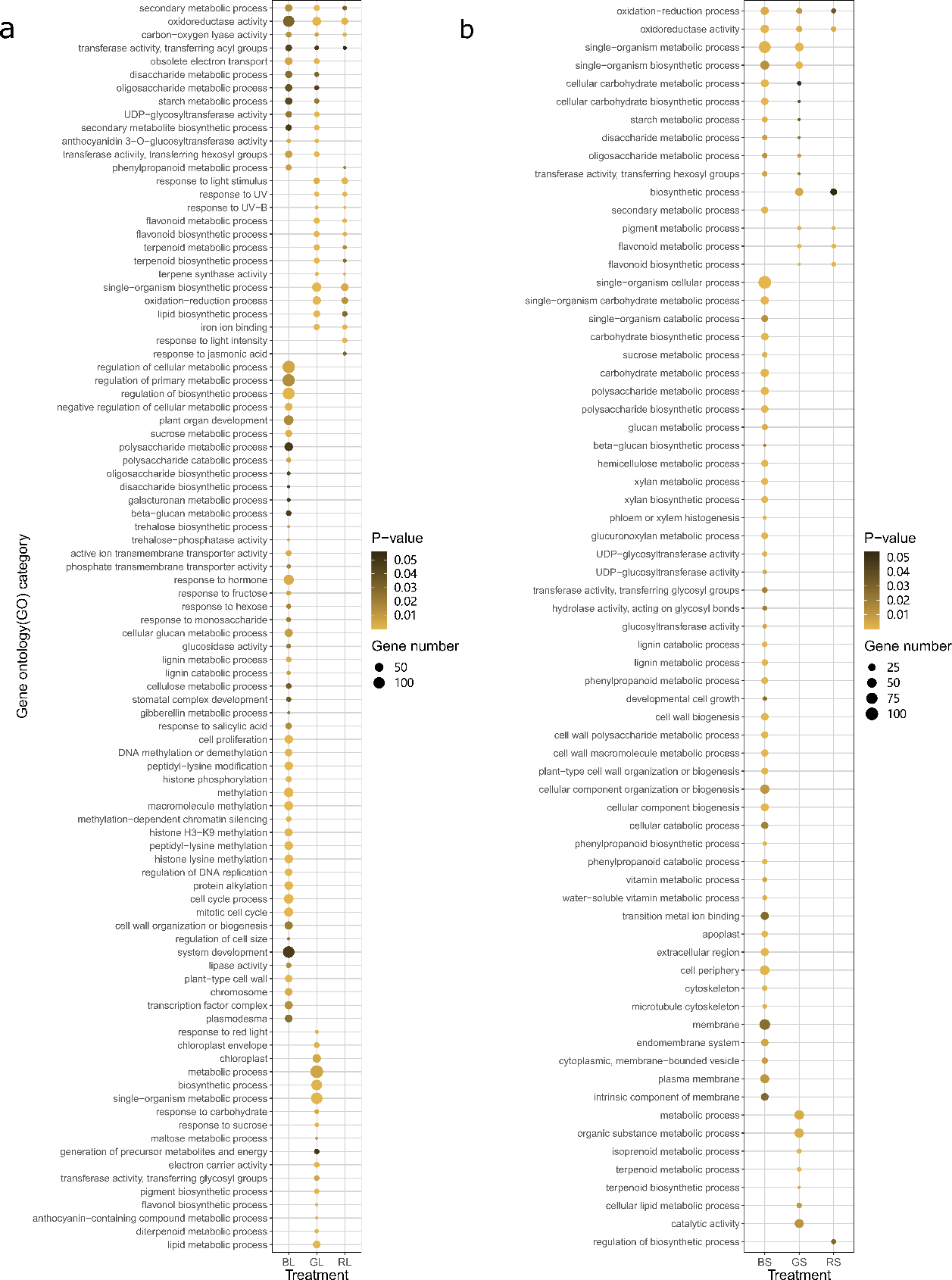


**Supplementary Fig. 6 GO functional enrichment of down-regulated in leaf (a) and stem (b) in response to monochromatic lights.** The color scale indicates the significance of enrichment and the circle size indicates the number of genes enriched in the term.


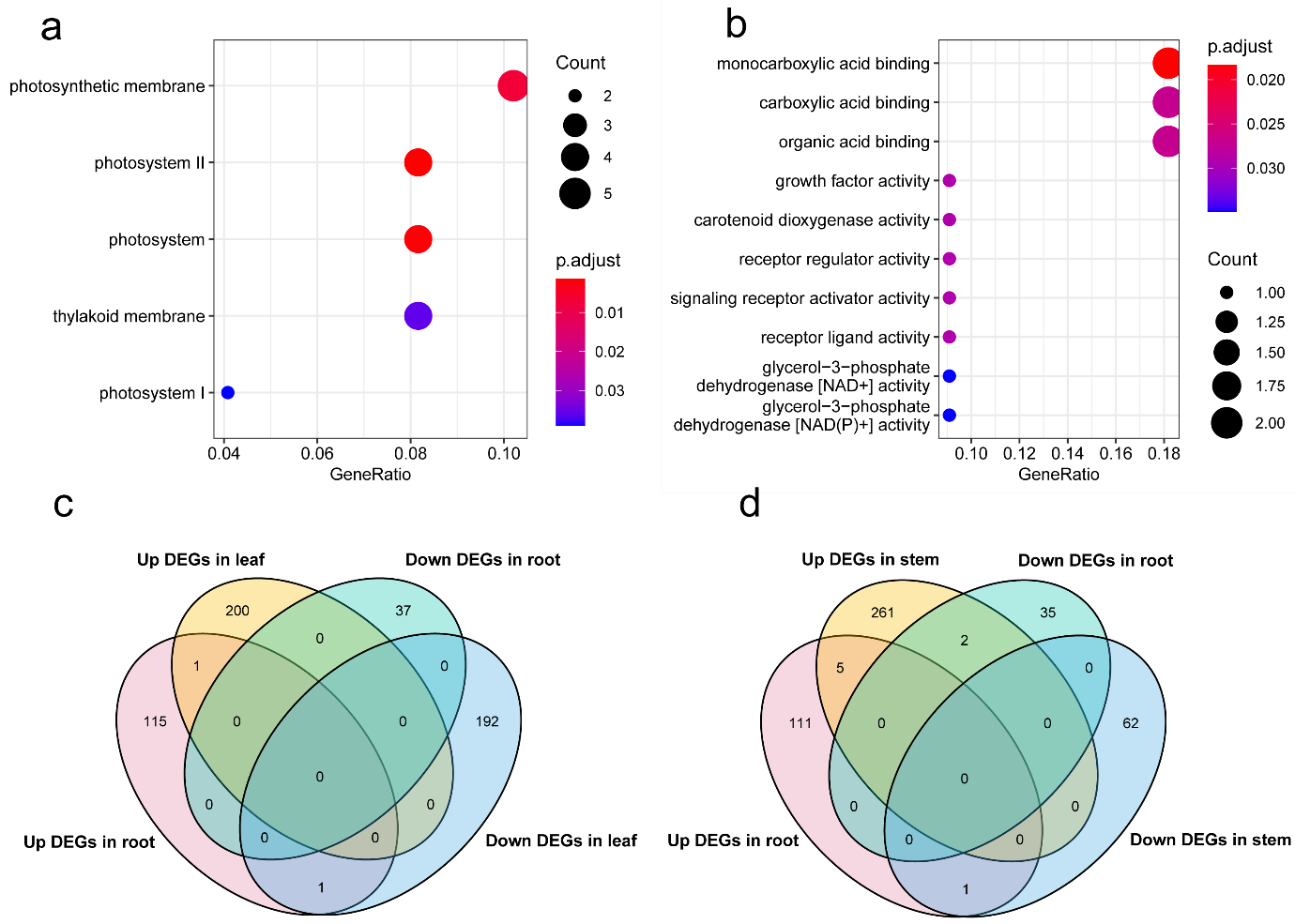


**Supplementary Fig. 7 GO functional enrichment of up-regulated (a) and down-regulated (b) DEGs in root under green light.** The color scale indicates the significance of enrichment and the circle size indicates the number of genes enriched in the term.


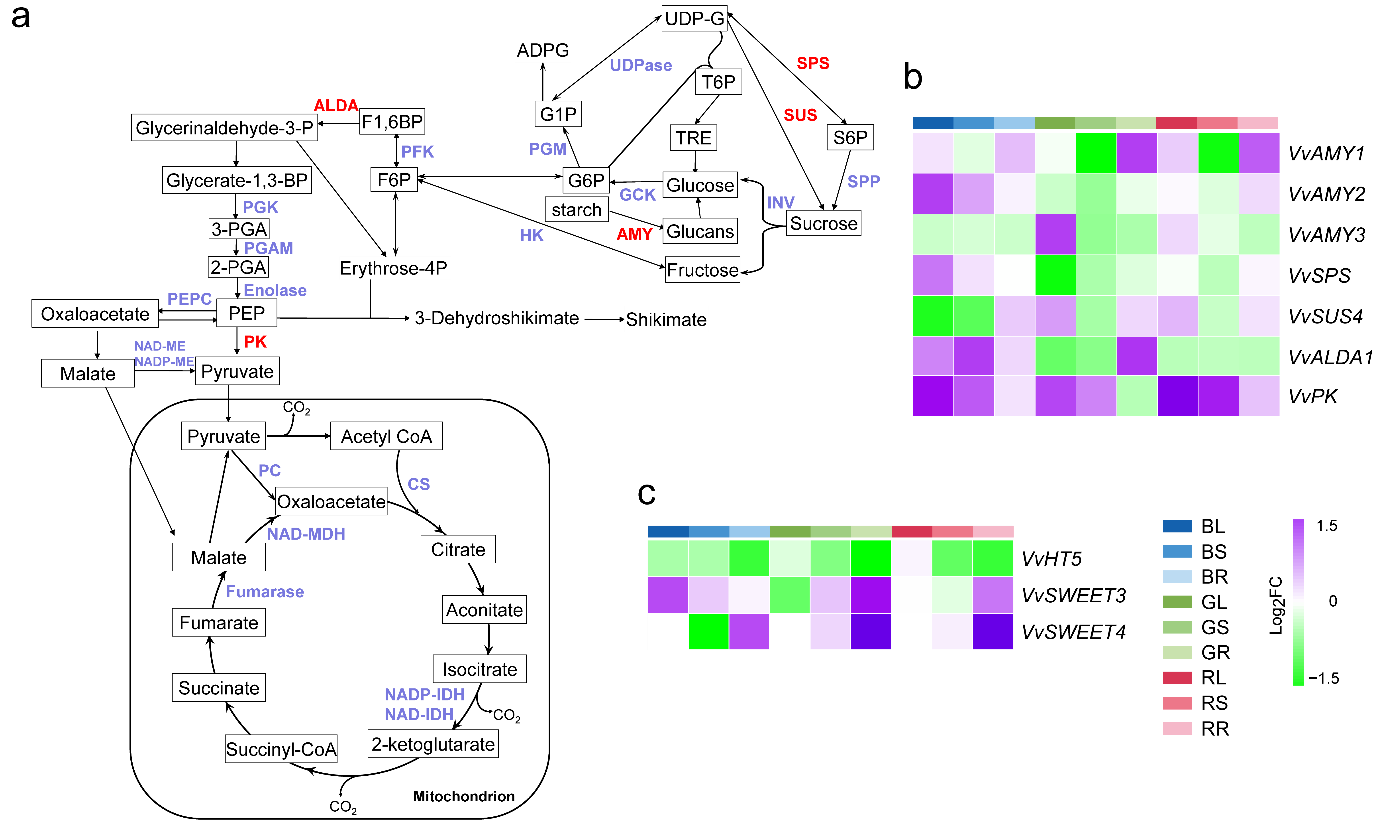


**Supplementary Fig. 8 DEGs for carbon metabolism and sugar transport under different monochromatic light treatments.** The central carbon metabolism pathway with DEGs highlighted with red color (a). Effect of light quality on the differential expression fold change of genes involved in the carbon metabolism pathway (b) and genes encoding sugar transporters (c). The color scale indicates the fold change of DEGs with purple up-regulation and green for down-regulation. B for blue light, R red light, G green light, W white light; L for leaf, S stem, and R root.


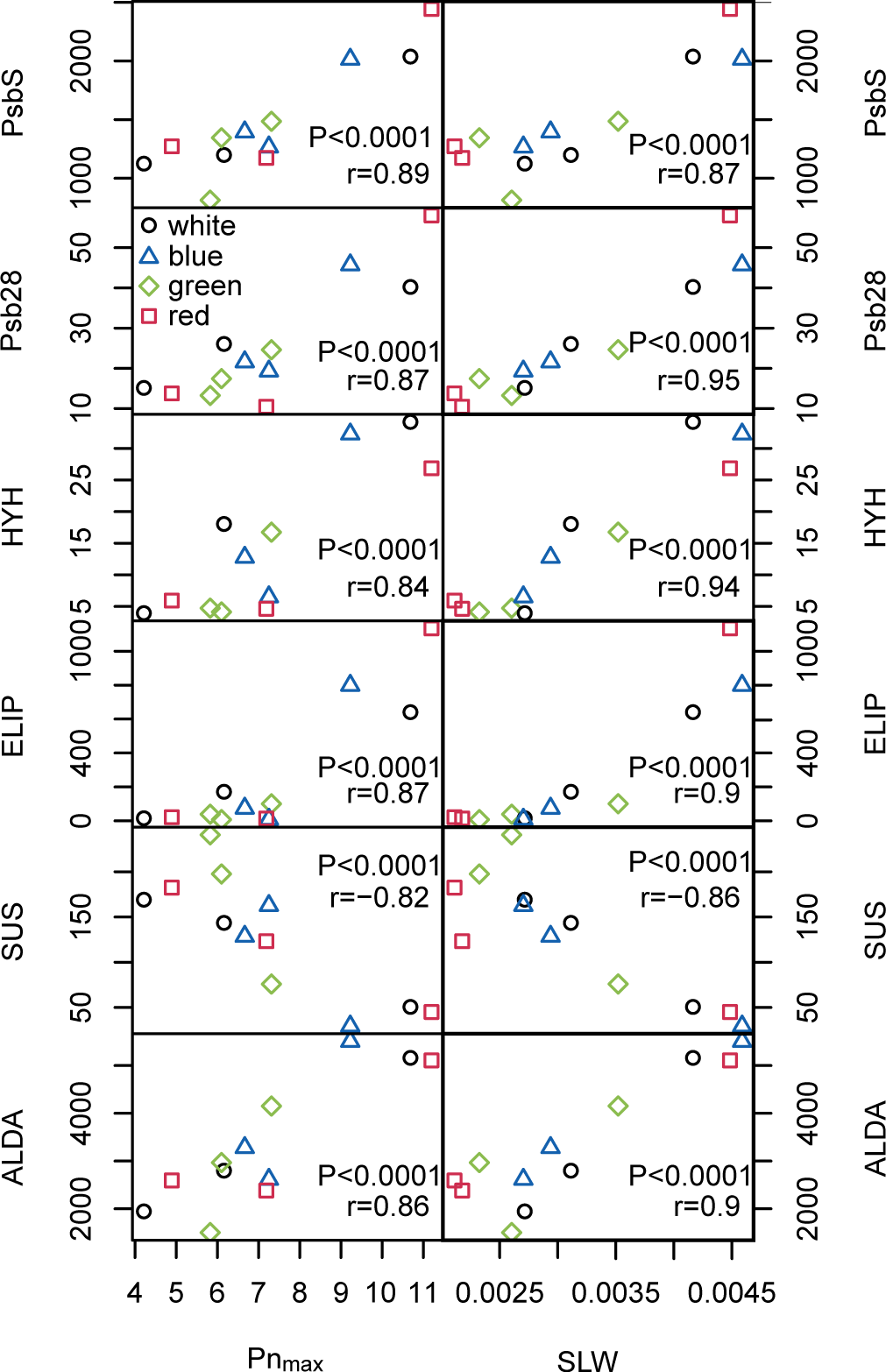


**Supplementary Figure.9 Correlation plot between traits and DEGs in leaf under different light qualities.** Pn_max_ and SLW indicate the light-saturated photosynthetic rate and specific leaf weight of leaves at range 7, respectively. The Pearson correlation coefficient and *P* values were indicated for each trait-gene pair.


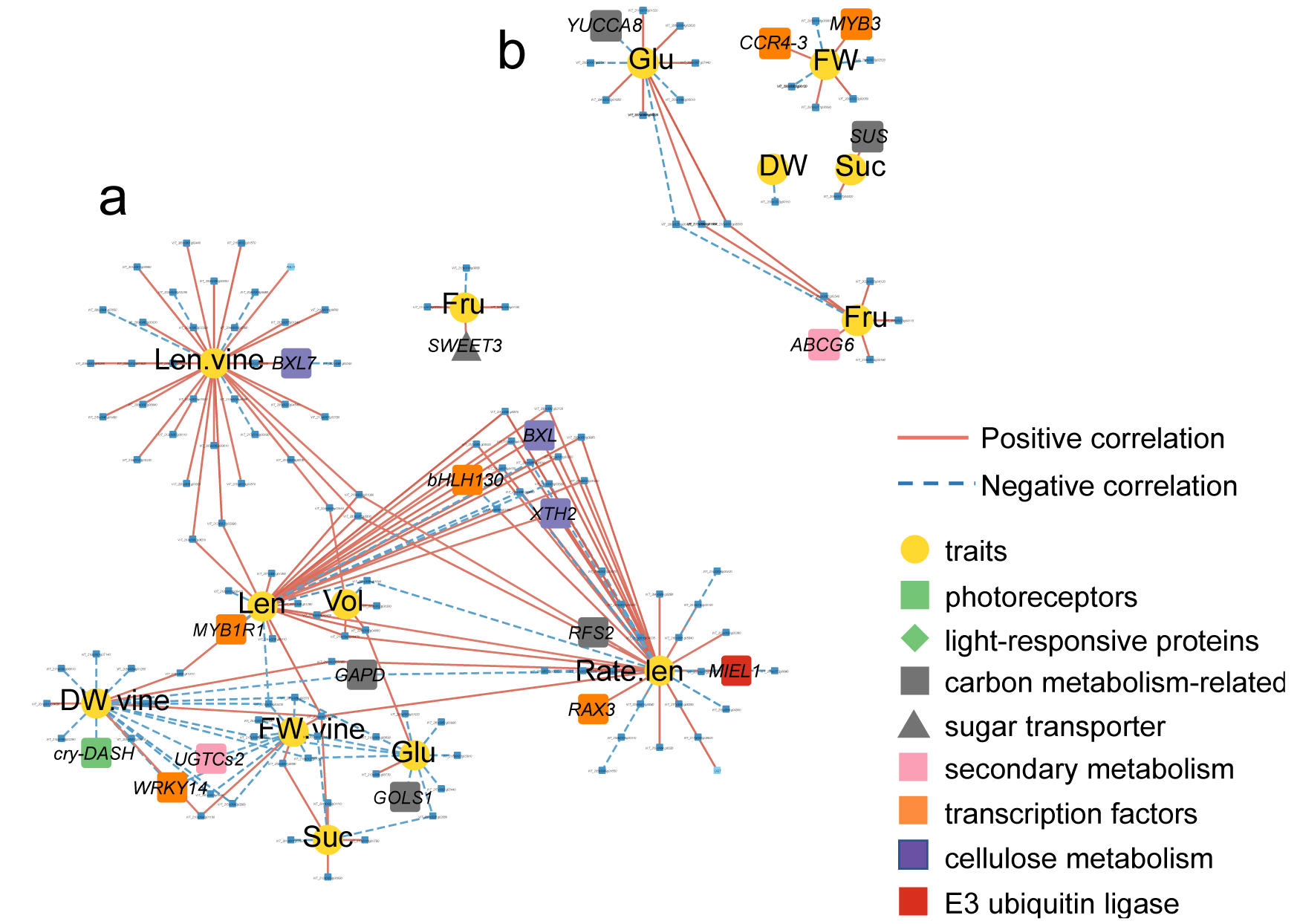


**Supplementary Figure.10 Correlation network between physiological and biochemical traits and DEGs in stem (a) and root (b) under different light qualities.** In (a), the Len, Vol, Glu, Fru, and Suc denote the length, volume, glucose content, fructose content and sucrose content of stem internod at range 7, respectively; Len. vine, FW. vine and DW. vine denote length, fresh weight, and dry weight of the whole stem, respectively; Rate.len.7 refers to the growth rate of stem internode at range 7. In (b), Glu, Fru, Suc, FW and DW denote glucose content, fructose content, sucrose content, fresh weight, and dry weight of the roots, respectively.
